# Establishment of *HRAS^G12V^* Transgenic Medaka as a Stable Tumor Model for *In Vivo* Screening of Anticancer Drugs

**DOI:** 10.1371/journal.pone.0054424

**Published:** 2013-01-14

**Authors:** Yuriko Matsuzaki, Haru Hosokai, Yukiyo Mizuguchi, Shoji Fukamachi, Atsushi Shimizu, Hideyuki Saya

**Affiliations:** 1 Division of Gene Regulation, Institute for Advanced Medical Research, School of Medicine, Keio University, Tokyo, Japan; 2 Division of Material and Biological Sciences, Japan Women's University, Tokyo, Japan; 3 Laboratory of Evolutionary Genetics, Department of Chemical and Biological Sciences, Japan Women's University, Tokyo, Japan; 4 Department of Molecular Biology, School of Medicine, Keio University, Tokyo, Japan; 5 Japan Science and Technology Agency, Core Research for Evolutional Science and Technology, Tokyo, Japan; Northwestern University Feinberg School of Medicine, United States of America

## Abstract

Most targeted anticancer drugs have been identified by screening at the molecular or cellular level *in vitro*. However, many compounds selected by such costly and time-consuming screening do not prove effective against tumors *in vivo*. The development of anticancer drugs would thus be facilitated by the availability of an *in vivo* screening system based on a multicellular organism. We have now established a transgenic line of the freshwater fish medaka in which melanophores (melanocytes) proliferate in a manner dependent on heat shock–induced signaling by a human RAS oncoprotein. The human *HRAS^G12V^* oncogene was expressed under the control of a melanophore-specific gene promoter in order to allow visualization of tumor growth in live fish maintained in a water tank. The expression of *HRAS^G12V^* was induced as a result of Cre-mediated recombination by exposure of the fish to a temperature of 37°C for 30 min, given that the Cre gene was placed under the control of a medaka heat shock promoter. One of the stable transgenic lines developed abnormal pigment cell proliferation in the eyes and epidermis with 100% penetrance by 6 months postfertilization. Sorafenib, an inhibitor of RAS signaling, was administered to the transgenic fish and was found both to reduce the extent of melanophore proliferation and to improve survival. The transgenic medaka established here thus represents a promising *in vivo* system with which to screen potential anticancer drugs that target RAS signaling, and this system can readily be adapted for the screening of agents that target other oncogenes.

## Introduction

Further improvements in cancer treatment will require the development of new molecularly targeted drugs that inhibit the growth and spread of tumors. Most such drugs developed to date were first identified by screening at the cell or molecular level and were subsequently tested in animals, usually mice, before being entered into clinical trials. Although progress in imaging technology has allowed evaluation of tumor growth in live experimental animals, the availability of a system that does not require such imaging would be expected to facilitate the testing of anticancer drugs.

The zebrafish is often adopted as a vertebrate model for chemical or genetic screening in part because it is readily raised in large numbers and its embryos are transparent [1], [2]. The medaka is a small egg-laying freshwater fish native to East Asia and has several advantages over zebrafish for such studies. The size of the medaka genome (∼800 Mb) is thus about half that of the zebrafish genome, with its draft sequence having been recently published [3], and medaka is able to survive in both cold and warm conditions. Medaka has also proved suitable for toxicology and carcinogenesis studies [4], [5]. In Organisation for Economic Co-operation and Development (OECD) test guidelines, the medaka fish was recommended as a model acute (from 1992) or prolonged (from 1984) toxicity test (http://www.oecd-ilibrary.org/environment/oecd-guidelines-for-the-testing-of-chemicals-section-2-effects-on-biotic-systems_20745761).

Mutations in the components of the RAS/RAF/MEK/ERK and RAS/PI3K/PTEN/AKT/mTOR signaling cascades have been detected in many types of human cancer [6]. Given that tumor cells become addicted to the activation of these signaling pathways [7], interruption of such oncogenic signaling has the potential to improve the prognosis of affected cancer patients. RAS and RAS-related signal transduction pathways are thus promising targets for inhibition of the growth of cancer cells. Several effective inhibitors of such signaling components have been examined in clinical trials. One such inhibitor, sorafenib, targets BRAF and has completed phase III clinical trials, having been found to be effective for the treatment of various cancer types including hepatocellular carcinoma, renal cell carcinoma, and melanoma [8]. On the other hand, no clinically effective agents that inhibit the GTP-bound active form of RAS, an upstream regulator of RAF, have been identified, although inhibitors have been developed that block the farnesylation of RAS, which allows the protein to bind to cell membranes [6]. The further development of inhibitors of RAS or RAS pathway components may thus yield new and effective anticancer drugs.

We have now developed a transgenic medaka tumor model suitable for the testing of potential inhibitors of oncogenic signaling. To construct this model, we made use of a constitutively active mutant of human HRAS, HRAS^G12V^, which has been detected in various tumors at a relatively high frequency. Transgenic zebrafish that express HRAS^G12V^ and develop melanoma [9] or that express KRAS^G12V^ and develop liver tumors [10] were recently established and found to be suitable tumor models for further study. In our medaka model, we made use of the Cre-*loxP* system and a medaka heat shock promoter to ensure that HRAS^G12V^ is expressed specifically in melanophores (melanocytes) and in a manner dependent on water temperature. The resulting transgenic fish developed readily visible melanoma-like tumors with 100% penetrance at 6 months postfertilization (mpf). We treated the fish with sorafenib as a test of their suitability for screening of drugs that target RAS signaling.

## Materials and Methods

### Medaka maintenance

Medaka (*Oryzias latipes*) (provided by NBRP Medaka, Aichi, Japan) was maintained according to established protocols (http://www.shigen.nig.ac.jp/medaka/medakabook). All animal experiments were performed in accordance with protocols approved by the Animal Care and Use Committee of Keio University (Permit Number: 12038-0).

### Plasmid construction

The medaka *tyr* promoter sequence [11](TYRO_ORYLA in http://medakagb.lab.nig.ac.jp/Oryzias_latipes) was retrieved from the bacterial artificial chromosome (BAC) clone Ola1-014A12(provided by NBRP Medaka) by the polymerase chain reaction (PCR) with a specific primer set containing restriction endonuclease (*Xho*I and *Eco*RI) sites (meTyr-p-F, 5′-AAACTCGAGTCTGACCAATCTCTTCTGGAAGGCCAGCTG-3′; meTyr-p-R, 5′-TATGAATTCAAGATTCACCCACATCTGTCCAGACCTCAG-3′). The 3.4-kb PCR product was digested with *Xho*I and *Eco*RI and was then inserted into *Xho*I- and *Eco*RI-digested pTIS10-d-loxGFP-OlBMP4 (Figure S1A). A *Not*I site of the resultant recombinant was replaced to *Bam*HI site using *Bam*HI-linker. The human HRAS^G12V^ cDNA sequence was retrieved from pMX-HRASV12-IRES-EGFP [12] by PCR with a specific primer set containing *Bam*HI sites (HRAS-F, 5′-ATATGGATCCATGACGGAATATAAGCTGGTGGTGGTGG-3′; HRAS-R1, 5′-ATGCGGATCCTCAGGAGAGCACACACTTGCAGCTCAT-3′). The 0.6-kb PCR product was digested with *Bam*HI and then inserted into the *Bam*HI-digested plasmid containing the medaka *tyr* promoter region to yield pTIS10-tyr-loxGFP-HRASV12. The inducible Cre expression plasmid pTIS9-hBhsp70creCherry (Figure S1B) contains the DNA sequence for a fusion protein of Cre recombinase and the red fluorescent protein mCherry under the control of the medaka *hsp70* promoter. This plasmid also contains the *TagBFP* gene which encodes blue fluorescent protein (Evrogen, Moscow, Russia) under the control of the promoter of a medaka embryonic globin (hemoglobin a-0) gene [13] as a marker for selection of transgenic lines. This marker allowed the selection of embryos harboring the *cre* transgene on the basis of the blue fluorescence of their blood without heat treatment. The sequences of all recombinant constructs were verified.

### Microinjection of plasmid DNA

We preferred to use a black wild-type medaka line for microinjection of plasmids so as to allow visualization of melanophore proliferation. The black wild-type strain HB11A(provided by NBRP Medaka) was difficult to maintain in our facility, however, so we crossed this line with the propagative H1bRHi line (orange-red variety) and selected highly pigmented offspring (HBK11). Fertilized eggs were obtained from this hybrid, and one-cell-stage embryos were injected with approximate 5 nl of a mixture containing 50 pg of recombinant DNA, 0.5× I-*Sce*I buffer, 0.025 U of I-*Sce*I, 0.5× Yamamoto's medium, and 0.05% phenol red. Embryos were raised and outcrossed with wild-type fish to identify potential transgenic animals.

### Extraction of genomic DNA

Genomic DNA was extracted from the fin of adults or from the whole body of larvae. The tissue samples were incubated for 10 min at room temperature in 200 μl of a lysis buffer containing 20 mM Tris-HCl (pH 8.0), 5 mM EDTA, 400 mM NaCl, 0.3% SDS, and proteinase K (200 μg/ml; Sigma-Aldrich, St. Louis, MO, USA), after which the lysates were heated for 5 min at 95°C and subjected directly to PCR.

### Selection of *Tg(tyr:HRAS^G12V^)/Tg(hsp:cre)* double-transgenic medaka

A homozygous *Tg(tyr:HRAS^G12V^)* transgenic strain was crossed with a heterozygous *Tg(hsp:cre)* strain, and resulting Cre-positive embryos were selected on the basis of the blue fluorescence of their blood.

### Heat treatment

*Tg(tyr:HRAS^G12V^)/Tg(hsp:cre)* double-transgenic medaka at 1 or 4 week postfertilization (wpf) was subjected to heat treatment by incubation at 37°C for 30 minutes. The medaka *hsp70* promoter used for construction of the Cre plasmid also responds to stressors other than heat treatment, but this was not a problem for the present study. We studied both double-transgenic medaka showing melanophore hyperplasia without heat treatment as well as fish in which such hyperplasia was induced by heat treatment at 1 or 4 wpf.

### Extraction of total RNA and RT

Total RNA was isolated from various medaka tissues with the use of an RNeasy Mini Kit (Qiagen, Hilden, Germany) and with RNase-Free DNase Set (Qiagen) to eliminate genomic DNA from the samples. The extracted RNA was subjected to reverse transcription (RT) with the use of a Transcriptor First Strand cDNA Synthesis Kit (Roche Applied Science, Rotkreuz, Switzerland).

### PCR assay conditions

The presence of the recombination-specific sequence in *Tg(tyr:HRAS^G12V^)* medaka was confirmed by PCR with a forward primer targeted to the enhanced green fluorescent protein (EGFP) gene (EGFP-F, 5′-AGCTGGACGGCGACGTAAACGG-3′) and a reverse primer targeted to human *HRAS* (HRAS-R2, 5′-ACCACCACCAGCTTATATTCCGTC-3′). The amplification protocol included an initial incubation at 95°C for 10 min; 35 cycles of 94°C for 20 s, 57°C for 30 s, and 72°C for 40 s; and a final incubation at 72°C for 6 min. The reaction mixture contained 10 μl of 2× Ampdirect plus (Shimadzu, Kyoto, Japan), 0.5 μl of genomic DNA, 0.5 μM of each primer, and 0.5 U of BIOTAQ HS DNA Polymerase (Bioline, London, UK) in a total volume of 20 μl. The presence of human *HRAS* cDNA among RT products was determined with a human *HRAS* forward primer (HRAS-F, 5′-AGCTGGACGGCGACGTAAACGG-3′) and a human *HRAS* reverse primer (HRAS-R2). Intact medaka *hras* cDNA was amplified as a positive control with the primers Ol-HRAS-F (5′-ATTGGCCCGGTCCTACGGTA-3′) and Ol-HRAS-R (5′-GCGACAGCTCATGCAGTCCTGA-3′). Medaka cytoplasmic actin mRNA was examined as an RNA quality control (OlCA1-F, 5′-GGGAAATTGTCCGTGACATC-3′; OlCA1-R, 5′-GACTCATCGTACTCCTGCTT-3′). The amplification protocol for human *HRAS* included an initial incubation at 95°C for 2 min; 35 cycles of 95°C for 10 s, 57°C for 20 s, and 72°C for 20 s; and a final incubation at 72°C for 6 min. The amplification protocol for medaka *hras* comprised an initial incubation at 95°C for 2 min; 35 cycles of 95°C for 10 s, 55°C for 20 s, and 72°C for 20 s; and a final incubation at 72°C for 6 min. The amplification protocol for medaka cytoplasmic actin cDNA included an initial incubation at 95°C for 2 min; 30 cycles of 95°C for 10 s, 57°C for 20 s, and 72°C for 30 s; and a final incubation at 72°C for 6 min. These latter three reaction mixtures contained 2 μl of 10×LA buffer (Takara, Kyoto, Japan), 0.5 μl of cDNA, 0.5 μM of each primer, and 0.5 U of LA Taq DNA Polymerase (Takara) in a total volume of 20 μl.

### Histochemical staining

The entire fish body was fixed in 4% paraformaldehyde, embedded in paraffin, serially sectioned, and stained with hematoxylin-eosin. Histochemical images were captured with a Biorevo BZ-9000 microscope (Keyence, Osaka, Japan).

### Drug administration

For protocol 1 of drug administration, *Tg(tyr:HRAS^G12V^)/Tg(hsp:cre)* double-transgenic medaka manifesting abnormal proliferation of melanophores at 4 mpf were divided into two groups and either sorafenib (BAY-43-9006; Cayman Chemical, Michigan, USA)or dimethyl sulfoxide (DMSO) vehicle was added to the water at a final concentration of 0.1 μM and 0.1%, respectively. The fish were exposed to these agents for four 24-h periods over 2 weeks. For protocol 2, fish at 2 mpf were divided into two groups and either sorafenib or DMSO was added to the water at 0.3 μM or 0.1%, respectively. The fish were exposed to these agents for 24-h periods four times over 4 weeks. The geometric mean of the maximum plasma concentration (*C*_max_) of sorafenib is ∼5 mg/l (∼10 μM) in patients taking the drug orally [14], and the median inhibitory concentration of sorafenib for wild-type BRAF is 22 nM [15]. In preliminary experiments, we found that administration of 4 μM sorafenib for 48 h was lethal to medaka, whereas three of five fish died after administration of 2 μM sorafenib five times for 48 h over the course of a month. We therefore selected sorafenib concentrations of 0.1 and 0.3 μM for our experiments. Fish were photographed before and after drug administration.

### Photography

Fish were photographed with a Leica DFC300FX camera, and images were processed with Leica Application Suite version 2.7.1.R1 software (Leica Microsystems, Wetzlar, Germany). The black color associated with melanophore hyperplasia in the fish body was quantified with the use of LAS-3000 Mini and Multi Gauge version 3.0 software (Fujifilm, Tokyo, Japan).

### Statistical analysis

Differences in mean melanophore hyperplasia and in survival were evaluated with Student's *t* test and the log-rank test, respectively. A *P* value of <0.05 was considered statistically significant.

## Results

### Transgenic medaka expressing human *HRAS^G12V^*

**Figure 1:**
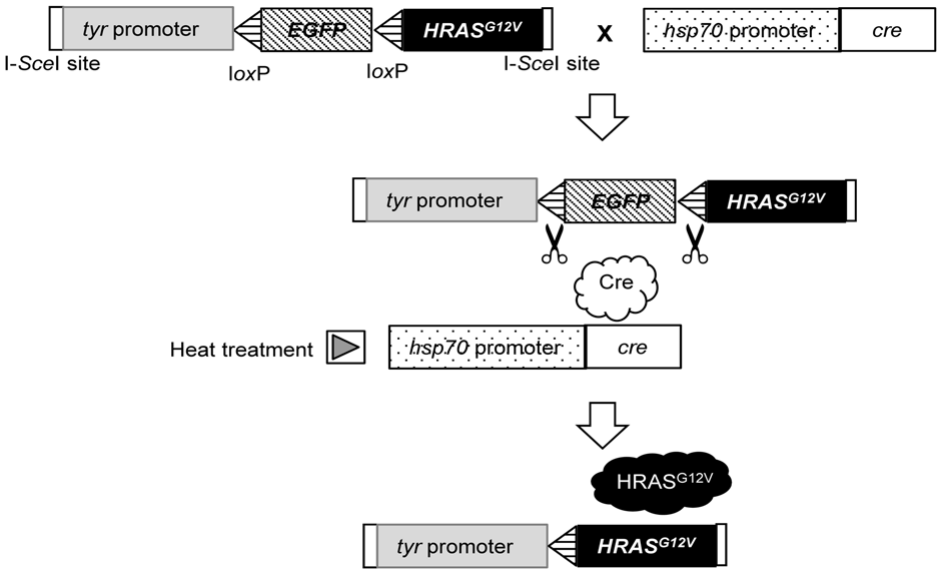
Generation of transgenic medaka expressing human *HRAS^G12V^*. A plasmid containing the human *HRAS^G12V^* cDNA downstream of the medaka tyrosinase gene (*tyr*) promoter as well as the *EGFP* gene positioned between *loxP* sequences was constructed for the generation of transgenic medaka. These transgenic fish were crossed with another transgenic line harboring the gene for Cre recombinase (*cre*) under the control of the medaka *hsp70* promoter. The resulting double-transgenic animals express Cre recombinase after exposure to heat shock, resulting in excision of the *EGFP* gene and expression of *HRAS^G12V^*.

We first attempted to establish a transgenic medaka line constitutively expressing human *HRAS^G12V^* under the control of the promoter for the medaka tyrosinase gene (*tyr*), which is expressed specifically in cells having melanosome such as melanophores and retinal pigment epithelial cells, but the animals died before achieving adulthood. We therefore developed a conditional gene expression system in which *HRAS^G12V^* is expressed in response to the induction of Cre recombinase by heat shock (Figure 1). The transgene vector pTIS10-tyr-loxGFP-HRASV12 was thus designed to express *HRAS^G12V^* under the control of the *tyr* promoter in response to Cre-mediated recombination. The *cre* gene was placed under the control of the medaka *hsp70* (*heat shock protein 70*) gene promoter. Cre-mediated recombination removes the EGFP gene from the *HRAS^G12V^* construct, allowing expression of the human *HRAS^G12V^* to be induced when the fish are exposed to a temperature of 37°C for 30 min. The Cre protein irreversibly excises nucleotides contain sequences of the EGFP after the heat treatment, and the human *HRAS^G12V^* is constitutively expressed.

The plasmid pTIS10-tyr-loxGFP-HRASV12 was injected into ∼200 embryos at the one-cell stage, which were then raised to adult fish. The resulting mature animals were outcrossed with wild-type fish to detect DNA fragments derived from pTIS10-tyr-loxGFP-HRASV12. We obtained four stable transgenic lines harboring the *HRAS^G12V^* construct. These four *Tg(tyr:HRAS^G12V^)* strains were crossed with the *Tg(hsp:cre)* strain (harboring the *cre* gene under the control of the medaka *hsp70* promoter). The resulting *HRAS^G12V^* heterozygotes harboring *cre* were expected to express *HRAS^G12V^* in response to heat treatment. However, the heat shock element in medaka is activated not only by heat but also by various other environmental stressors such as ammonia, nitrate, nitrite, a change in pH, or the proliferation of bacteria, mold, or parasites in the water tank. We therefore maintained the *Tg(tyr:HRAS^G12V^)* and *Tg(hsp:cre)* strains independently, and the two strains were crossed only when offspring were required for study.

The double-transgenic offspring resulting from crosses between each of the four *Tg(tyr:HRAS^G12V^)* lines and the *Tg(hsp:cre)* strain manifested abnormal proliferation of melanophores in the eyes and epidermis with different frequencies after heat treatment (Table 1). The prevalence of melanophore proliferative lesions (MPLs) at 6 mpf (with heat treatment applied at 4 wpf) thus ranged from 12.5 to 100%. These differences in penetrance may be attributable to differences in *HRAS^G12V^* expression level resulting from differences in transgene copy number or insertion sites. All subsequent experiments were performed with offspring of *Tg(tyr:HRAS^G12V^)* line 1 and *Tg(hsp:cre)*, which developed MPLs with 100% penetrance at 6 mpf.

**Table 1:** Penetrance of melanophore proliferative lesions (MPLs) among *Tg(tyr:HRAS^G12V^)*/*Tg(hsp:cre)* double-transgenic medaka derived from four different *Tg(tyr:HRAS^G12V^)* lines (Tg lines 1 to 4).

	Tg line 1	Tg line 2	Tg line 3	Tg line 4
Total number observed	16	11	12	8
Number with MPLs	16	6	5	1
Penetrance (%)	100	54.5	41.7	12.5

The fish were subjected to heat treatment by incubation at 37°C for 30 minutes at 4 wpf and examined at 6 mpf.

**Figure 2:**
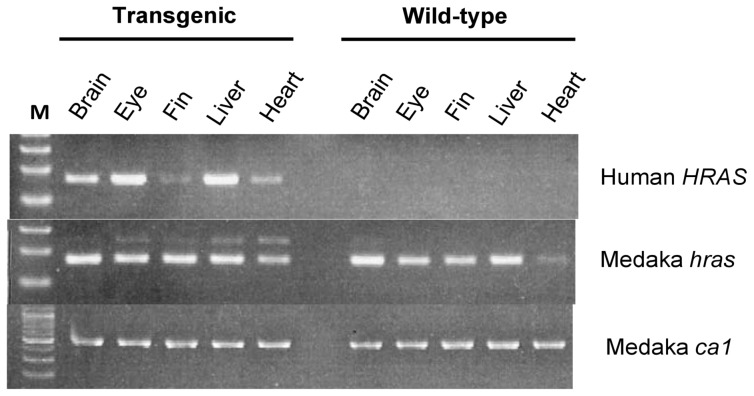
Double-transgenic medaka expresses human *HRAS^G12V^*. Total RNA isolated from the indicated tissues both of *Tg(tyr:HRAS^G12V^)*/*Tg(hsp:cre)* double-transgenic medaka at 8 mpf (with heat treatment at 4 wpf) and of wild-type medaka was subjected to RT-PCR analysis of human *HRAS* and medaka *hras*. Transcripts of the medaka cytoplasmic actin gene (*ca1*) were examined as an internal control of RNA quality. Lane M, DNA size markers.

We examined expression of *HRAS^G12V^* in the double-transgenic medaka at 8 mpf (with heat treatment at 4 wpf) by RT-PCR analysis (Figure 2). Expression of *HRAS^G12V^* was detected prominently in brain, eye, and liver as well as to a lesser extent in fin and heart of *Tg(tyr:HRAS^G12V^)*/*Tg(hsp:cre)* medaka but not in wild-type fish.

### Characteristics of *Tg(tyr:HRAS^G12V^)*/*Tg(hsp:cre)* double-transgenic fish

**Figure 3:**
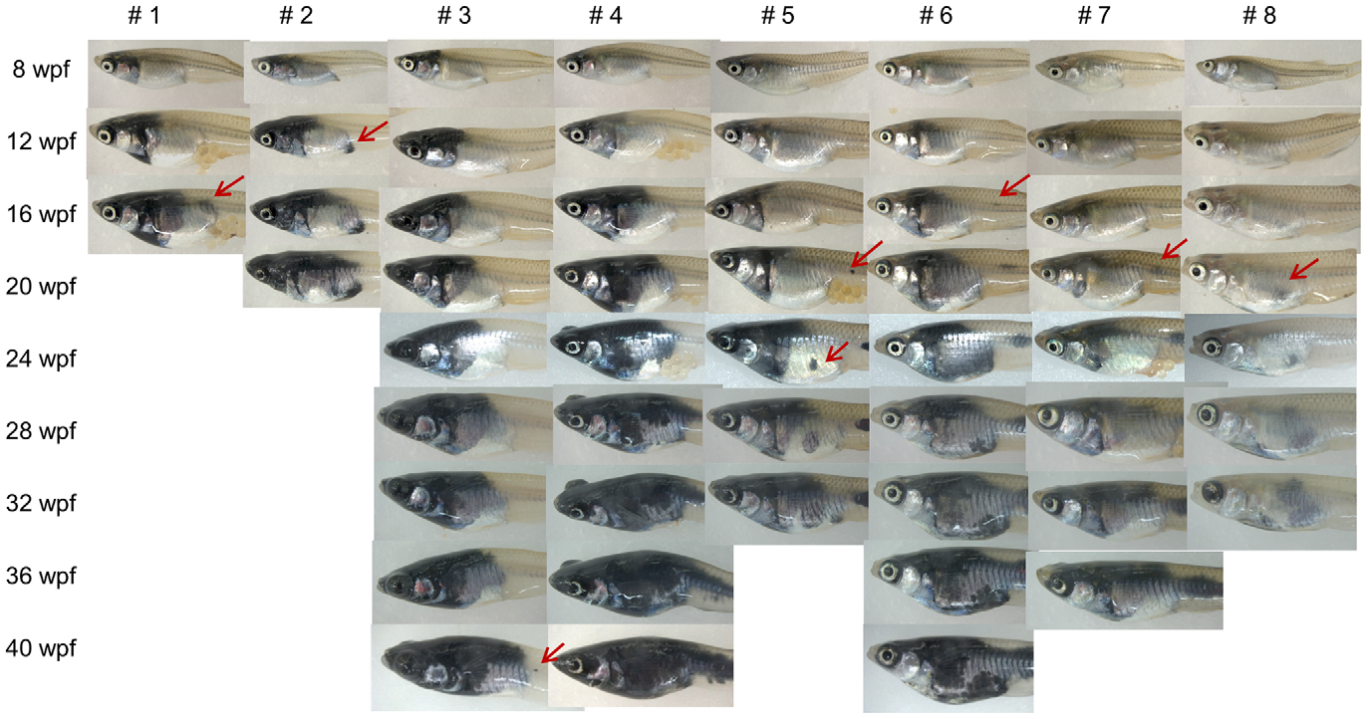
Characteristics of *Tg* *(* *tyr:HRAS^G12V^* *)*/*Tg* *(* *hsp:cre* *)* medaka. Age-dependent changes in eight double-transgenic fish were monitored after heat treatment at 1 wpf. Five of the eight fish manifested hyperproliferation of pigment cells around the eyes by 8 wpf. All of the fish had developed MPLs with characteristics of infiltrative tumors by 20 wpf, and all had died by 43 wpf. Arrows indicate the first appearance of metastasis-like lesions.

**Figure 4:**
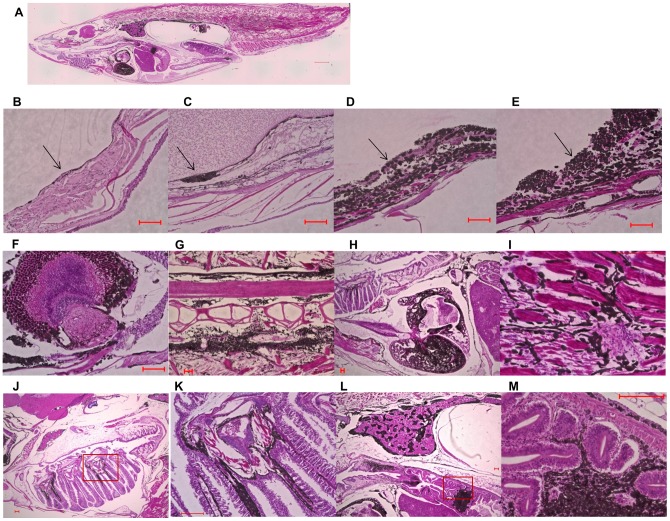
Hematoxylin-eosin staining of *Tg* *(* *tyr:HRAS^G12V^* *)*/*Tg* *(* *hsp:cre* *)* medaka. (**A**) Overall image of fish #7 in Figure 3. (**B**)– (**E**), Melanophore layers (arrows) of a wild-type adult fish (**B**) as well as of fish #7 (**C**), #6 (**D**), and #4 (**E**) in Figure 3. (**F**)– (**J**), Eye, bone and spinal cord, heart, muscle, and gill, respectively, of fish #7 in Figure 3. (**K**) Enlarged image of the boxed region in (**J**). (**L**) Kidney and digestive duct of fish #7 in Figure 3. (**M**) Enlarged image of the boxed region in (**L**). Scale bars: 1 mm (**A**) and 0.1 mm (**B**–**M**).

Among eight *Tg(tyr:HRAS^G12V^)/Tg(hsp:cre)* double-transgenic medaka born on the same day and subjected to heat treatment at 1 wpf, five fish manifested hyperproliferation of pigment cells around the eyes by 8 wpf (Figure 3). All of the fish had developed melanophore-derived infiltrative tumors by 20 wpf, with one animal that developed large MPLs having died. Small ectopic lesions potentially reflecting metastases of the primary lesions were also observed in seven fish (Figure 3). Melanophore infiltration became accelerated after 28 wpf, with all animals having died by 43 wpf (the average additional life span of these transgenic medaka survived more than 28 wpf was 9.2±0.90 wpf). The external surface of the fish manifested large black areas corresponding to melanophores in the skin, eyes, gills, abdomen, and bones. Hematoxylin-eosin staining also revealed abnormal proliferation of melanophores in the transgenic fish (Figure 4). Consistent with the fact that melanin synthesis normally takes place in the eyes (retinal pigment epithelium) and the melanophore layer that covers internal organs, the abnormal proliferation was apparent predominantly around the eyes and in the abdomen. The melanophore layers of the transgenic fish were thicker and manifested abnormal proliferation (Figure 4 C–E) compared with those of wild-type animals (Figure 4 B). The transgenic medaka also variously manifested the presence of an infiltrative mass in the eye (Figure 4 F) as well as melanophore infiltration into vertebrae, heart, muscle, gill, kidney, and the digestive duct (Figure 4 G–M). Cells of an atrium and ventricle of one animal were mostly replaced by melanophores (Figure 4 H). A magnified image of the digestive duct of one fish also showed melanophore infiltration into the stroma around the duct (Figure 4 M). These various pathological findings are similar to those of invasive melanoma in humans.

### MPL incidence and survival in *Tg*(*tyr:HRAS^G12V^*)/*Tg*(*hsp:cre*) medaka

**Figure 5:**
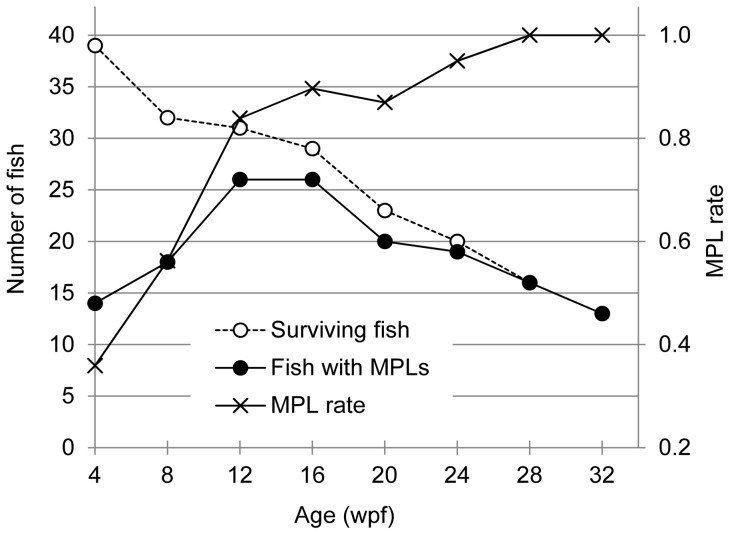
Age dependence of MPL incidence and survival in *Tg* *(* *tyr:HRAS^G12V^* *)*/*Tg* *(* *hsp:cre* *)* medaka. The transgenic fish were subjected to heat treatment at 1 wpf and then monitored for the development of MPLs and death. A single monitoring was performed with 39 *Tg(tyr:HRAS^G12V^)*/*Tg(hsp:cre)* medaka at 4 wpf.

Both *Tg(tyr:HRAS^G12V^)*/*Tg(hsp:cre)* and *Tg(hsp:cre)* medaka had a survival rate of <50% between the fertilized egg stage and 4 wpf when they were subjected to heat treatment at 1 wpf, whereas the corresponding value for wild-type fish was >80%. This finding suggested that Cre expression may have a toxic effect on the fish. We therefore decided to examine double-transgenic fish that survived beyond 4 wpf. Less that 40% of *Tg(tyr:HRAS^G12V^)/Tg(hsp:cre)* medaka manifested MPLs at 4 wpf, whereas >80% of the fish had developed MPLs at 12 wpf (Figure 5). The death rate increased after 16 wpf, and all of the fish had developed MPLs by 28 wpf. These findings suggested that the double-transgenic medaka developed MPLs by 6 mpf were suitable for screening of antitumor drugs that inhibit RAS signaling.

### Treatment of double-transgenic fish with sorafenib

**Figure 6:**
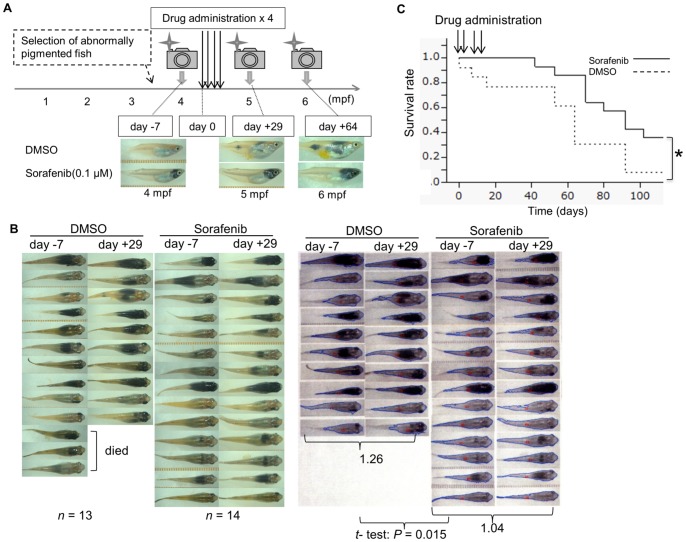
Effect of sorafenib (0.1 μM) treatment on melanophore hyperplasia and overall survival in *Tg* *(* *tyr:HRAS^G12V^* *)* */Tg* *(* *hsp:cre* *)* medaka. (**A**) Schedule of drug administration. (**B**) Photos taken from the dorsal side of all fish at 7 days before (day –7) and 29 days after (day +29) the first drug administration. The area of MPLs in the fish body was measured based on the set of captured images on the right, and the average fold change from day –7 to day +29 was calculated for each group. (**C**) Kaplan-Meier survival curves for the sorafenib-treated and control (DMSO-treated) groups were generated from the experiment shown in (**B**). Fish with obvious melanophore hyperplasia were divided into two groups, one of which was treated with 0.1 μM sorafenib (*n* = 14) and the other with DMSO as a control (*n* = 13). *P = 0.0267 (log-rank test).

Given that *Tg(tyr:HRAS^G12V^)* homozygotes have a low viability, we were not able to use them as parents for the generation of offspring for large-scale screening. Instead, we used the offspring of *Tg(tyr:HRAS^G12V^)*/*Tg(hsp:cre)* intracrosses and subjected them to heat treatment at 4 wpf. Fish with obvious melanophore hyperplasia were selected at 4 mpf and were divided into two groups, one of which was treated with 0.1 μM sorafenib (*n* = 14) and the other with DMSO as a control (*n* = 13) (Figure 6 A). Sorafenib is a multikinase inhibitor that targets BRAF, c-KIT receptor, VEGFR and PDGFR. Among them, it has substantial activity to inhibit BRAF, a downstream kinase of RAS.

The animals were photographed 7 days before (day –7) and 29 days after (day +29) the first drug administration. Three fish of the control group died before day +29, whereas all of the sorafenib-treated fish survived.

We quantified the black areas caused by melanophore hyperplasia in the fish body by image analysis and calculated the fold change from day –7 to day +29 (Figure 6 B), obtaining average values of 1.04 for the 14 sorafenib-treated fish and 1.26 for the 10 control fish *(P* = 0.015, Student's *t* test). Furthermore, the survival rate for the sorafenib-treated group was significantly greater (*P* = 0.0267, log-rank test) than that for the control group (Figure 6 C). At 110 days after the first drug administration, the survival rate for the sorafenib-treated group was thus 35.7% (5/14), whereas that for the control group was only 7.7% (1/13). We performed a second trial with fish at 2 mpf treated with 0.3 μM sorafenib (*n* = 15) or DMSO *(n* = 15) (Figure S2 A, B) and calculated the fold change in melanophore hyperplasia between day –4 and day +31, obtaining values of 1.65 and 1.79, respectively *(P* = 0.066). At 170 days after the first drug administration, the survival rate for the sorafenib-treated group was 33.3% (5/15), whereas that for the control group was 0% (0/15), a statistically significant difference (*P* = 0.0484) (Figure S2 C). These results thus showed that sorafenib inhibited MPL growth and increased survival time in the double-transgenic medaka.

## Discussion

We obtained four transgenic medaka lines, *Tg(tyr:HRAS^G12V^)*, that harbor human *HRAS^G12V^*, which encodes a constitutively active mutant of HRAS. The *Tg(tyr:HRAS^G12V^)/Tg(hsp:cre)* double-transgenic animals derived from a cross between *Tg(tyr:HRAS^G12V^)* and *Tg(hsp:cre)* medaka express *HRAS^G12V^* in response to heat treatment.

The promoter of zebrafish *mitf* (microphthalmia transcription factor gene) has often been used to control the expression of human oncogenes such as *HRAS* or *BRAF* in melanocytes in transgenic zebrafish models [16], [17], [18], [19]. However, *mitf* gene is expressed in cell types other than melanocytes, the expression of oncogenes under the *mitf g*ene promoter may cause diverse toxic effects on the fishes. We therefore chose the medaka *tyr* promoter for our model of melanophore hyperplasia, given that tyrosinase is a key enzyme in the melanin biosynthetic pathway, catalyzing the conversion of tyrosine to dopaquinone. The *tyr* gene is expressed specifically during melanocyte differentiation, and the promoter of the mouse *tyr* gene has been used to achieve melanocyte-specific expression of human oncogenes in several mouse models [20]. Given that constitutive expression of active HRAS under the control of *tyr* gene promoter resulted in poor viability of the transgenic medaka due to toxicity caused by mutant HRAS, we employed a heat shock inducible system. An inducible *KRAS^G12V^* transgenic zebrafish model for liver tumorigenesis was previously reported [10]. However, since their system requires a steroid (mifepristone) to induce the *KRAS^G12V^*gene, the drug screening is done in the presence of mifepristone and, thereby, the effects are based on the combination of a candidate drug and mifepristone. Our system using a heat shock-inducible melanophore-specific expression of *HRAS^G12V^* has an advantage over those previous model systems for drug screening. Our transgenic medaka may not be sufficient for a preclinical model for cancer therapy but provide a good tool to assess the efficacy of selected candidate drugs.

Larvae of *Tg(hsp:cre)* medaka showed a high mortality rate, with >50% of the animals dying before 4 wpf. Cre toxicity has been described previously [21], [22], with Cre expression having been found to inhibit the proliferation of cultured cells as well as to induce numerous chromosomal aberrations or sister chromatid exchanges. These effects are thought to result from DNA damage caused by the recombinase activity of Cre at cryptic *loxP* sites in the genome. The *Tg(hsp:cre)* medaka established in the present study should thus be preserved in the form of frozen sperm in order to prevent the accumulation of DNA damage.

We performed image analysis to quantify the black areas corresponding to melanophore hyperplasia in the double-transgenic medaka. Such analysis revealed that the extent of MPLs was significantly reduced by sorafenib treatment, and this effect was associated with a significant increase in overall survival. Although we cannot rule out the possibility that the photographs analyzed do not accurately reflect the actual extent of melanophore hyperplasia, we believe that this approach is reliable. The tumor-like black tissue composed of proliferating melanophores appeared in a manner dependent on *HRAS^G12V^* activation and expanded with age. The *Tg(tyr:HRAS^G12V^)/Tg(hsp:cre)* medaka established in the present study thus represents a promising model system for the screening of inhibitors of RAS signaling *in vivo*. Given that MPL expansion was found to be dependent on RAS activation and that melanophores are readily observed by visual inspection of live fish, our system is potentially applicable to the development of *in vivo* tumor models based on the activation of other oncogenes.

The cause of death for transgenic medaka is not obvious. But, it is possible that the reduction of feeding activity is a main cause because there was marked infiltration of tumor-like cells into digestive tract of the dead medaka. Our data showed that sorafenib inhibited MPL growth and increased survival time in the double-transgenic medaka. However, it should be noted that the tumors are in the medaka background and, thereby, it could impact therapeutic response. In addition, the effect of sorafenib on MPL expansion in our second protocol of drug administration (Figure S2) was not as marked as that in the first protocol (Figure 6). Sorafenib has substantial activity to inhibit BRAF, a downstream kinase of RAS, and it therefore might not be expected to block the RAS/PI3K/PTEN/AKT/mTOR signaling pathway. Tumor growth observed in the presence of sorafenib might thus be due to signaling by this latter pathway in an adaptive response. The combination of an inhibitor of RAS/PI3K/PTEN/AKT/mTOR signaling with sorafenib might thus be expected to be more effective for inhibition of tumor growth than sorafenib alone.

Finally, in addition to the potential of *Tg(tyr:HRAS^G12V^)/Tg(hsp:cre)* medaka for *in vivo* screening of potential anticancer drugs that target RAS signaling, the establishment of a melanoma-like cell line from these animals may prove useful for studies on the mechanism of *HRAS*-dependent tumorigenesis *in vitro*.

## Supporting Information

Figure S1 **Map of plasmids used.** (**A**) pTIS10-d-loxGFP-OlBMP4: The promoter of medaka *tyr* was inserted between the *Xho*I and *Eco*RI recognition sites replacing the promoter of the medaka desmin gene, and the human *HRAS^G12V^* cDNA sequence was inserted between the *Bam*HI and *Not*I recognition sites replacing the medaka *bmp4* sequence. (**B**) pTIS9-hBhsp70creCherry: The DNA sequence for a fusion protein of Cre recombinase and the red fluorescent protein mCherry under the control of the medaka *hsp70* promoter. This plasmid also contains the TagBFP gene under the control of the promoter of a medaka embryonic globin gene.
(TIF)

Figure S2 **Effect of sorafenib** (**0.3 μM**) **treatment on melanophore hyperplasia and overall survival in** ***Tg*** *(* ***tyr:HRAS^G12V^*** *)* **/** ***Tg*** *(* ***hsp:cre*** *)* **medaka.** (**A**) Schedule of drug administration. (**B**) Photos taken from the dorsal side of all fish at 4 days before (day –4) and 31 days after (day +31) the first drug administration. The area of MPLs in the fish body was measured based on the set of captured images on the right, and the average fold change from day –4 to day +31 was calculated for each group. (**C**) Kaplan-Meier survival curves for the sorafenib-treated and control (DMSO-treated) groups were generated from the experiment shown in (**B**). Fish with obvious melanophore hyperplasia were divided into two groups, one of which was treated with 0.3 μM sorafenib (*n* = 15) and the other with DMSO as a control (*n* = 15). *P = 0.0484 (log-rank test).
(TIF)
