# Supplementary figures and images for: Establishment of HRASG12V Transgenic Medaka as a Stable Tumor Model for In Vivo Screening of Anticancer Drugs
Source: PLoS One. 2013 Jan 14;8(1):e54424. doi: 10.1371/journal.pone.0054424 (PMC3544794; doi:10.1371/journal.pone.0054424)

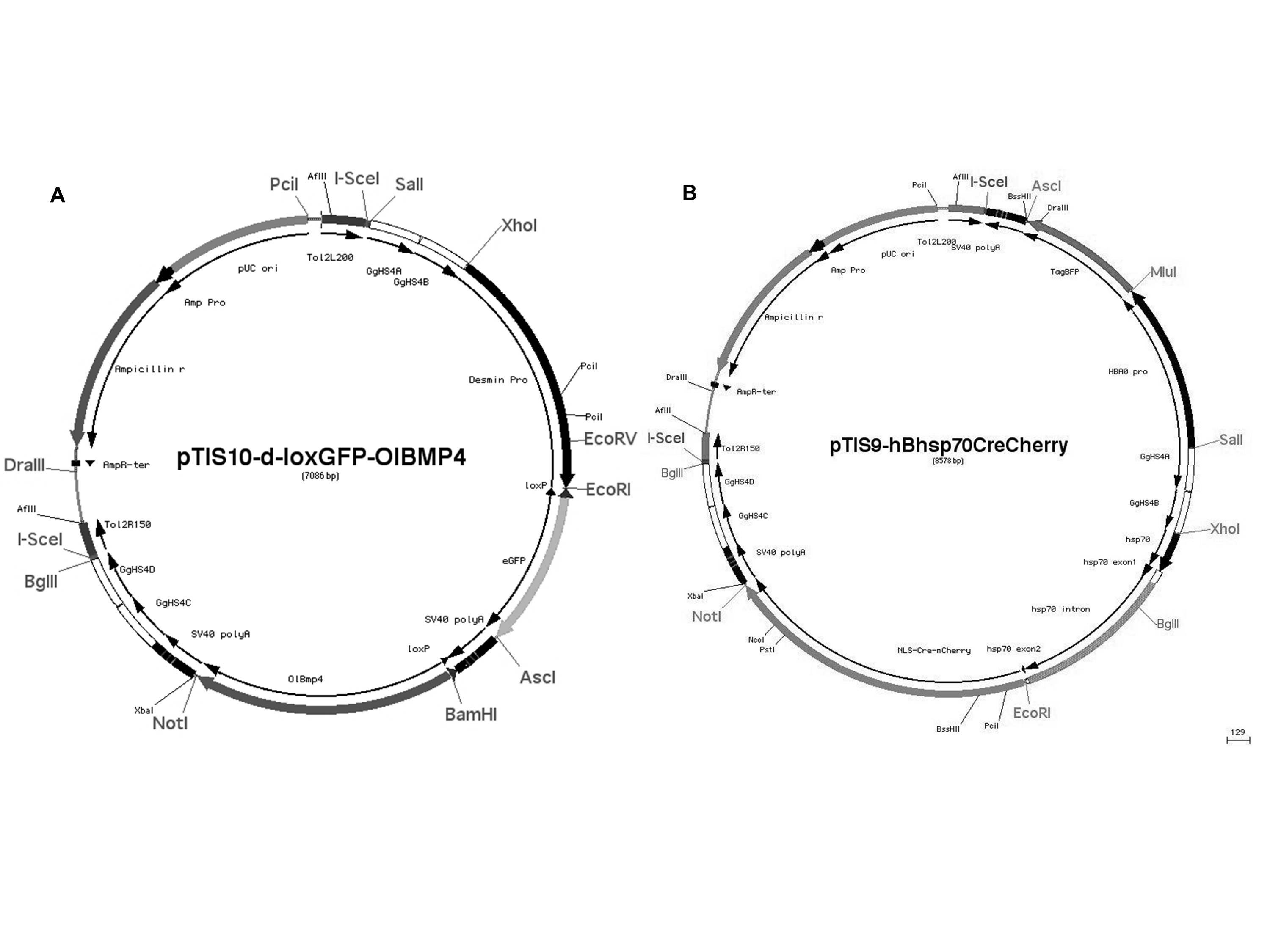

Supplement: Figure S1 — Map of plasmids used. (A) pTIS10-d-loxGFP-OlBMP4: The promoter of medaka tyr was inserted between the XhoI and EcoRI recognition sites replacing the promoter of the medaka desmin gene, and the human HRASG12V cDNA sequence was inserted between the BamHI and NotI recognition sites replacing the medaka bmp4 sequence. (B) pTIS9-hBhsp70creCherry: The DNA sequence for a fusion protein of Cre recombinase and the red fluorescent protein mCherry under the control of the medaka hsp70 promoter. This plasmid also contains the TagBFP gene under the control of the promoter of a medaka embryonic globin gene. (TIF) [file pone.0054424.s001.tif]

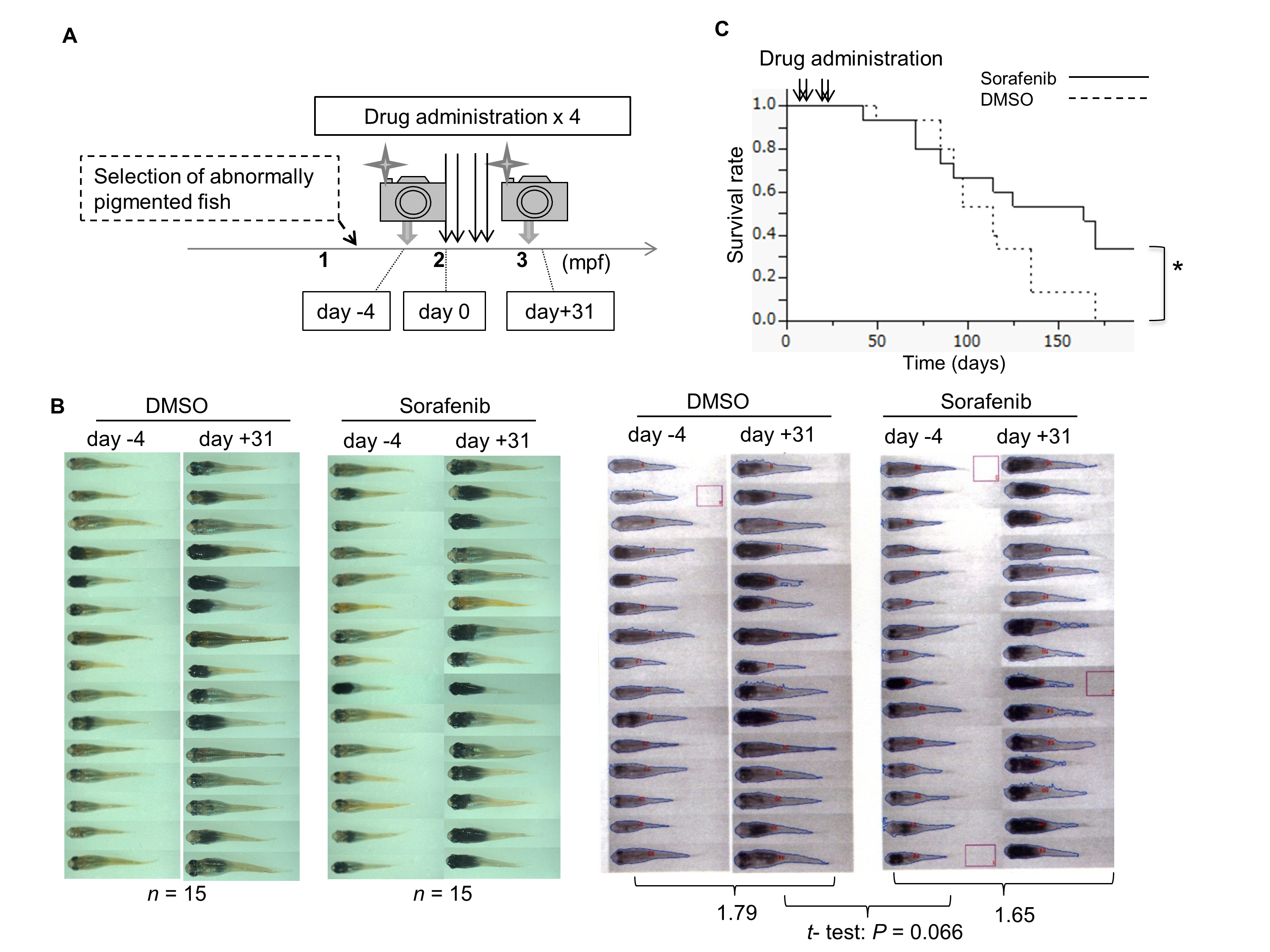

Supplement: Figure S2 — Effect of sorafenib (0.3 μM) treatment on melanophore hyperplasia and overall survival in Tg ( tyr:HRASG12V ) / Tg ( hsp:cre ) medaka. (A) Schedule of drug administration. (B) Photos taken from the dorsal side of all fish at 4 days before (day –4) and 31 days after (day +31) the first drug administration. The area of MPLs in the fish body was measured based on the set of captured images on the right, and the average fold change from day –4 to day +31 was calculated for each group. (C) Kaplan-Meier survival curves for the sorafenib-treated and control (DMSO-treated) groups were generated from the experiment shown in (B). Fish with obvious melanophore hyperplasia were divided into two groups, one of which was treated with 0.3 μM sorafenib (n = 15) and the other with DMSO as a control (n = 15). *P = 0.0484 (log-rank test). (TIF) [file pone.0054424.s002.tif]
